# Progress and Future Trends in PET/CT and PET/MRI Molecular Imaging Approaches for Breast Cancer

**DOI:** 10.3389/fonc.2020.01301

**Published:** 2020-08-12

**Authors:** Yue Ming, Nan Wu, Tianyi Qian, Xiao Li, David Q. Wan, Caiying Li, Yalun Li, Zhihong Wu, Xiang Wang, Jiaqi Liu, Ning Wu

**Affiliations:** ^1^PET-CT Center, National Cancer Center/National Clinical Research Center for Cancer/Cancer Hospital, Chinese Academy of Medical Sciences and Peking Union Medical College, Beijing, China; ^2^Department of Orthopedic Surgery, Peking Union Medical College Hospital, Peking Union Medical College and Chinese Academy of Medical Sciences, Beijing, China; ^3^Beijing Key Laboratory for Genetic Research of Skeletal Deformity, Beijing, China; ^4^Key Laboratory of Big Data for Spinal Deformities, Chinese Academy of Medical Sciences, Beijing, China; ^5^Department of Breast Surgical Oncology, National Cancer Center/National Clinical Research Center for Cancer/Cancer Hospital, Chinese Academy of Medical Sciences and Peking Union Medical College, Beijing, China; ^6^Department of Radiology, Peking Union Medical College Hospital, Peking Union Medical College and Chinese Academy of Medical Sciences, Beijing, China; ^7^Department of Diagnostic and Interventional Imaging, McGovern Medical School, Health and Science Center at Houston, University of Texas, Houston, TX, United States; ^8^Department of Medical Imaging, Second Hospital of Hebei Medical University, Hebei, China; ^9^Department of Breast Surgery, The Affiliated Yantai Yuhuangding Hospital of Qingdao University, Yantai, China; ^10^Department of Central Laboratory, Peking Union Medical College Hospital, Peking Union Medical College and Chinese Academy of Medical Sciences, Beijing, China

**Keywords:** breast cancer, PET, PET/CT, PET/MR, molecular imaging

## Abstract

Breast cancer is a major disease with high morbidity and mortality in women worldwide. Increased use of imaging biomarkers has been shown to add more information with clinical utility in the detection and evaluation of breast cancer. To date, numerous studies related to PET-based imaging in breast cancer have been published. Here, we review available studies on the clinical utility of different PET-based molecular imaging methods in breast cancer diagnosis, staging, distant-metastasis detection, therapeutic and prognostic prediction, and evaluation of therapeutic responses. For primary breast cancer, PET/MRI performed similarly to MRI but better than PET/CT. PET/CT and PET/MRI both have higher sensitivity than MRI in the detection of axillary and extra-axillary nodal metastases. For distant metastases, PET/CT has better performance in the detection of lung metastasis, while PET/MRI performs better in the liver and bone. Additionally, PET/CT is superior in terms of monitoring local recurrence. The progress in novel radiotracers and PET radiomics presents opportunities to reclassify tumors by combining their fine anatomical features with molecular characteristics and develop a beneficial pathway from bench to bedside to predict the treatment response and prognosis of breast cancer. However, further investigation is still needed before application of these modalities in clinical practice. In conclusion, PET-based imaging is not suitable for early-stage breast cancer, but it adds value in identifying regional nodal disease and distant metastases as an adjuvant to standard diagnostic imaging. Recent advances in imaging techniques would further widen the comprehensive and convergent applications of PET approaches in the clinical management of breast cancer.

## Introduction

**Figure 1:**
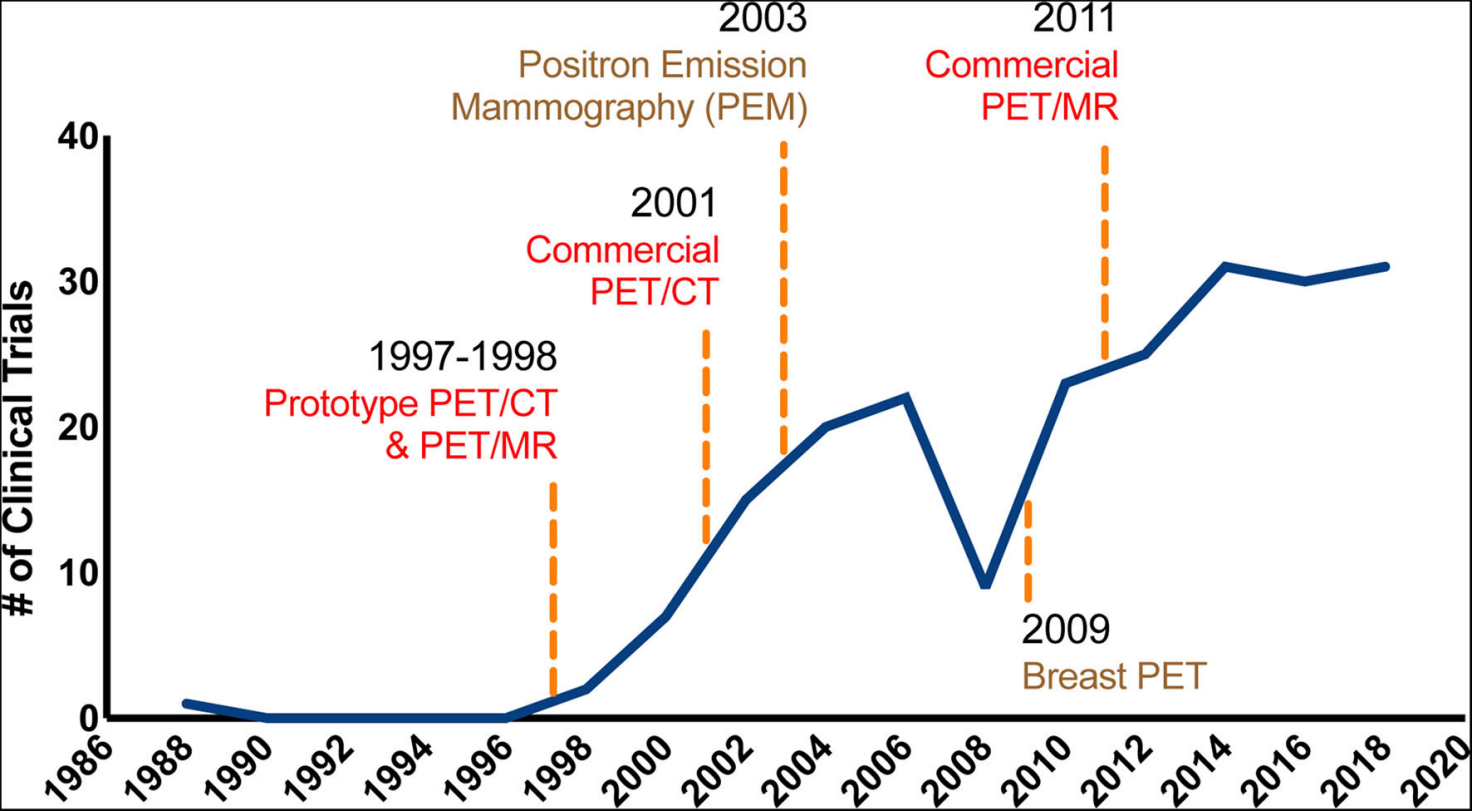
The developing utility of PET-based techniques in breast cancer. The graph represents a timeline of important technological developments in PET-based techniques. The solid line represents the change in the number of PubMed clinical trial publications for PET and breast cancer per year between 1988 and 2018. The earliest models of PET/MR and PET/CT date back to 1987 and 1988, respectively. As shown in the figure, the use of PET in breast cancer has increased dramatically over the past years, benefiting from the development of commercial equipment and breast-specific imaging approaches.

Breast cancer is the most common cancer and has the second highest cancer-related morbidity in women worldwide (1). Early diagnosis followed by timely treatment can significantly improve the survival of breast cancer patients. Modern clinical guidelines and standard treatments for breast cancer are based on accurate pathological diagnosis, clinical staging, and molecular subtyping (2). Mammography, ultrasound, and breast magnetic resonance imaging (MRI) are the primary methods of evaluating the local disease extent and stage. Computed tomography (CT) of the chest and abdomen, bone scans, and MRI are used to screen and stage distant metastases. However, the efficacy of clinical methods, including serum antigen protein markers and radiological imaging, is insufficient for breast cancer diagnosis and staging (3, 4). As a useful tool for the *in vivo* assaying of the metabolic characteristics of breast cancer (5, 6), positron-emission tomography (PET) using radioactive tracer ^18^F-fluorodeoxyglucose (^18^F-FDG) has been introduced as an additional imaging modality for these patients, facilitating breast cancer staging, distant-metastasis detection, prognostic prediction, and especially evaluation of the pathological response to treatment (7, 8). Combining PET with traditional radiological methodologies, including CT, MRI, and mammography, has shown a range of clinical utility for appropriate breast cancer patients. Several clinical trials and technical advances were achieved between 1988 and 2018 in testing the clinical utility of PET-based techniques in breast cancer (Figure 1). The application of PET in breast cancer has increased dramatically over the past years, benefiting from the development of commercial equipment and breast-specific imaging approaches.

In this review, we first revealed the biological basis of the fluorodeoxyglucose PET imaging in illustrating breast cancer. Then, multiple PET-based imaging techniques, including the breast-specific PET imaging, PET/CT, and PET/MRI, and their clinical applications were described in detail. The clinical applications started with the role in diagnosis and staging, including primary tumor, lymph node, and distant metastases, then local recurrence and therapeutic response evaluation. Furthermore, the clinical applicability of using PET-based imaging in cancer screening in asymptomatic women who are at high risk for breast cancer was also discussed. At last, recent advances in novel PET tracers and PET-based radiomics were demonstrated with the potential prospect in the future.

## Fluorodeoxyglucose PET and Tumor Biology

The increased ^18^F-FDG avidity of malignant cells forms the basis of breast cancer imaging with ^18^F-FDG-PET (7, 9). However, the uptake of ^18^F-FDG is also affected by the density of breast tissue. Dense breast tissue highly uptakes ^18^F-FDG but at amounts still lower than in breast cancer (10, 11). The intensity and pattern of ^18^F-FDG uptake in breast lesions are directly related to the biological characteristics of the tumor, such as pathological type, aggressiveness, and molecular subtype. Invasive ductal carcinomas (IDCs) exhibit higher avidity for ^18^F-FDG than noninvasive ductal carcinomas (5, 8, 12, 13), and triple-negative breast cancers display greater ^18^F-FDG uptake levels than other molecular subtypes (14). In some breast cancer subtypes (luminal A), FDG uptake is physiologically influenced by both the features of the tumor and hormonal fluctuations during the menstrual cycle (15). In addition to the FDG uptake value, advanced imaging features such as histogram of FDG-PET can be used to separate molecular and histological subtypes (16, 17). It has also been reported that FDG-PET radiomics are associated with various tumor aspects (namely, Ki67, pCR after NAC and risk of recurrence) and may potentially become unique pretreatment biomarkers of diagnosis and prognosis (18).

The breast tumor detection rate via ^18^F-FDG-PET is determined by the tumor size and pathology grade. Although the highest sensitivity of breast cancer detection using ^18^F-FDG-PET is reported as 96% among all tumor sizes, the sensitivity for small tumors (<1 cm) is reported to be below 60% (12). Because of its low sensitivity, ^18^F-FDG-PET is not routinely used for early-stage breast cancer patients, but it serves as a powerful complement to other imaging methods for accurate clinical staging in patients with stage III and IV disease. It has been reported that the application of ^18^F-FDG-PET would cause changes in the planned treatment in one-third of patients with advanced breast cancer by identifying distant-metastasis sites missed by conventional imaging methods (19).

## PET-Based Imaging Techniques

### Breast-Specific PET Imaging

Since whole-body PET imaging is suboptimal for the detection of breast lesions, it is recommended that a prone position be used to assist in breast-specific PET imaging scans (20). Thus, dedicated PET scanning modalities for the breast, including positron emission mammography and breast PET, have been developed to further improve the spatial resolution (21–23). The PEM system comprises two planar or curved detectors that scan the breasts with mild compression. MacDonald et al. (22) showed that the reconstructed spatial resolution in the PEM system can reach 2.4 mm. The limitation of PEM lies in its acquisition geometry, which may affect the clear imaging of lesions at the edge of the camera (for example, in the chest wall) and introduce structural superimposition (22). The PEM can help reclassify suspicious calcifications detected on standard mammography with largely improved accuracy (95%) (24). Additionally, a PEM-CT system has been introduced to further improve the detection power of the PEM system. The PEM-CT system is able to scan the breast in an uncompressed manner and image suspected lesions in 3 dimensions (25). The other existing prototype, the breast PET system, which has been developed relatively recently (Figure 1), acquires high-resolution breast imaging using multiple rotating planar detectors. We deem that the breast PET approach might help overcome the limitations of PEM.

### PET/CT

In most clinical applications, PET is combined with a CT system (PET/CT) to replace PET alone for providing anatomical information useful for accurately interpreting PET signals. The earliest models of PET/CT and PET/MR systems date back to 1987 (Figure 1). Similar to PET, PET/CT is not routinely performed without a previous diagnosis because of its lack of sensitivity, which is considerably low for lobular carcinomas (26) and tumors <1 cm in length (27). According to the guidelines of the National Comprehensive Cancer Network, PET/CT should not be applied for early-stage breast cancer in the absence of symptoms and is not indicated in the staging of clinical stage I or II or operable stage III breast cancer. However, PET/CT is recommended alongside diagnostic CT for identifying unsuspected regional nodal disease and/or distant metastases and in situations where standard staging studies are equivocal or suspicious, especially in the setting of locally advanced or metastatic disease (28). The clinical application of the PET/CT system is described in detail later in the manuscript.

### PET/MRI

Dynamic contrast-enhanced magnetic resonance imaging (DCE-MRI) is both a morphological and functional imaging tool and is the most sensitive technique to date for breast cancer screening (29). DCE-MRI is frequently used in breast cancer staging and treatment monitoring (30). Integrated PET/MRI, which can acquire both metabolic data and high-contrast morphological images in a single exam, has recently been used more often in breast cancer patients. PET/MRI has been suggested as a means to improve diagnostic accuracy and decrease false-positive rates over those obtained with MR (31, 32). It has also demonstrated increased axillary nodal metastasis sensitivity (33, 34). Bitencourt et al. (35) reported that 100% sensitivity and 55% specificity could be achieved in breast cancer diagnosis using multiparametric PET/MRI evaluation (including DCE, diffusion-weighted imaging [DWI], and 3D ^1^H-MRSI). Pinker et al. (36) also reported that in their study of 78 indeterminate or suspicious lesions, half of the unnecessary biopsies would be performed with PET/MRI compared with conducting MRI alone. Furthermore, Jena et al. (37) indicated that by adding acquisition time into the pharmacokinetic parameters of PET/MRI, an accuracy of 94.50% can be achieved. The pharmacokinetic DCE-MRI parameters can be further correlated with metastatic status, Ki67 expression (38), and prognosis (39, 40). Catalano et al. demonstrated that ^18^F-FDG PET/MRI could distinguish between molecular subtypes in 13/21 breast cancer patients (41).

## Clinical Application of PET-Based Imaging Techniques

### Imaging the Primary Breast Cancer

**Figure 2:**
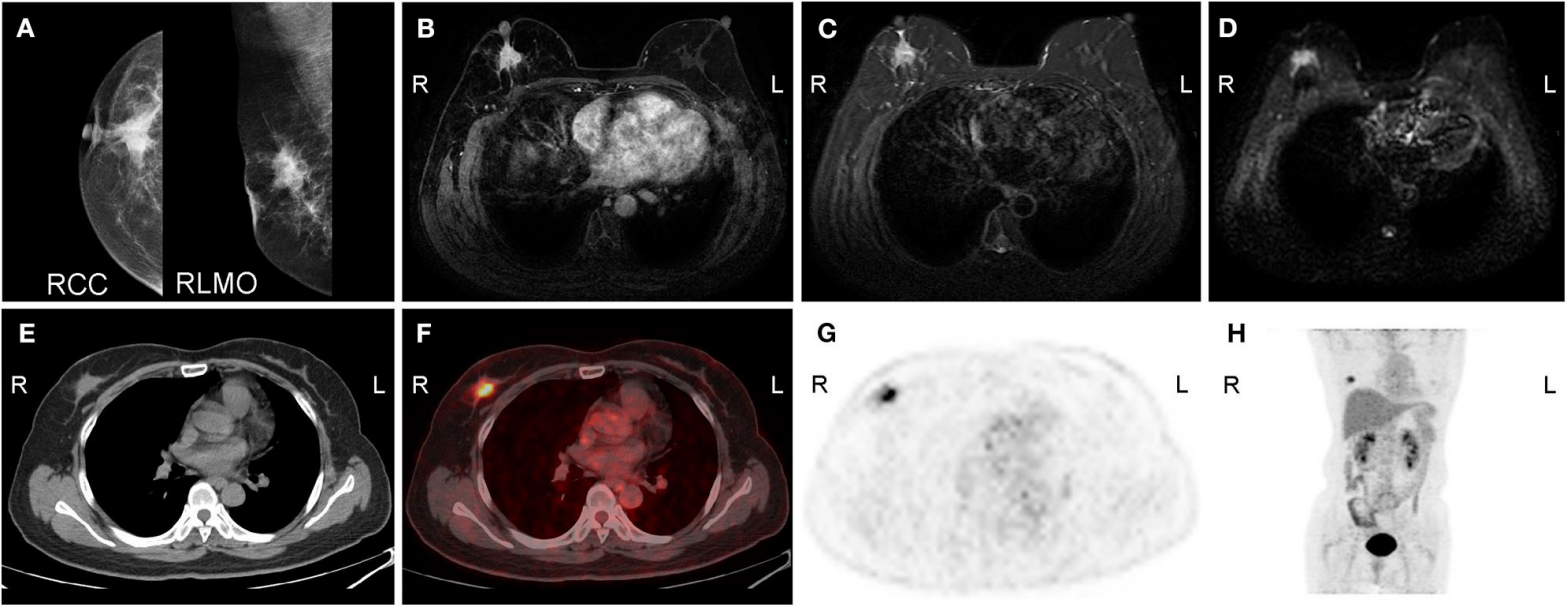
A 50-year-old woman with carcinoma of the right breast. **(A)** Molybdenum target mammography shows a mass in the upper quadrant of the right breast with an irregular shape, fuzzy boundary, speculation, linear calcification, and a retracted nipple. **(B)** Enhanced MRI shows that the right breast mass was significantly enhanced during the arterial phase, with a high signal on **(C)** T2WI/FS and **(D)** DWI. Axial **(E)** CT, **(F)** fused image, **(G)** FDG-PET, and **(H)** MIP (maximal intensity projection) image show irregular nodules with intense uptake on the upper outer quadrant of the right breast. R, right; L, left; RCC, right craniocaudal; RMLO, right mediolateral oblique.

Breast cancer detection, including assessment of tumor size and multifocality, is mainly based on mammography, ultrasound, and MRI. Several studies have reported that FDG-PET displays low sensitivity and relatively high specificity and accuracy in the detection of primary breast cancer (7, 12, 42, 43). Figure 2 shows multiple images of PET/CT, MRI, and mammography in a female patient with right breast cancer. Compared with MRI, PET/CT has been shown to be less sensitive but more specific in breast cancer detection (44). Jung et al. (26) compared the accuracy of PET/CT and MRI among 105 patients with biopsy-proven breast cancers, illustrating that MRI identified all 105 (100%) primary tumors, whereas PET/CT correctly detected 85 (81.0%) primary tumors. In the detection of multifocal breast cancers, Ergul et al. (45) reported that the sensitivity of PET/CT and MRI was 67 vs. 78%, while the specificity was 100 vs. 53%, respectively.

In addition, PET/MRI performs better in primary breast cancer detection than PET/CT does, but compared with the detection with MRI, the improvement is limited. In a study comparing MRI, PET/CT, and PET/MRI in 50 breast cancer patients, PET/MRI and MRI showed higher accuracy in identifying the tumor size than PET/CT [41/50 (82%) by using PET/MRI, 41/50 (82%) by using MRI, and 34/50 (68%) by using PET/CT; *P* < 0.05] (34). Additionally, PET/MRI and MRI also showed higher accuracy in identifying multifocal or multicentric breast cancer than PET/CT [8/9 (89%) by using both PET/MRI and MRI vs. 5/9 (56%) by using PET/CT; *P* < 0.05] (34). Thus, FDG-PET is not recommended in breast cancer screening and is utilized in primary tumor detection only when standard procedures deliver a suspicious result.

### Imaging the Lymph-Node Metastases

**Figure 3:**
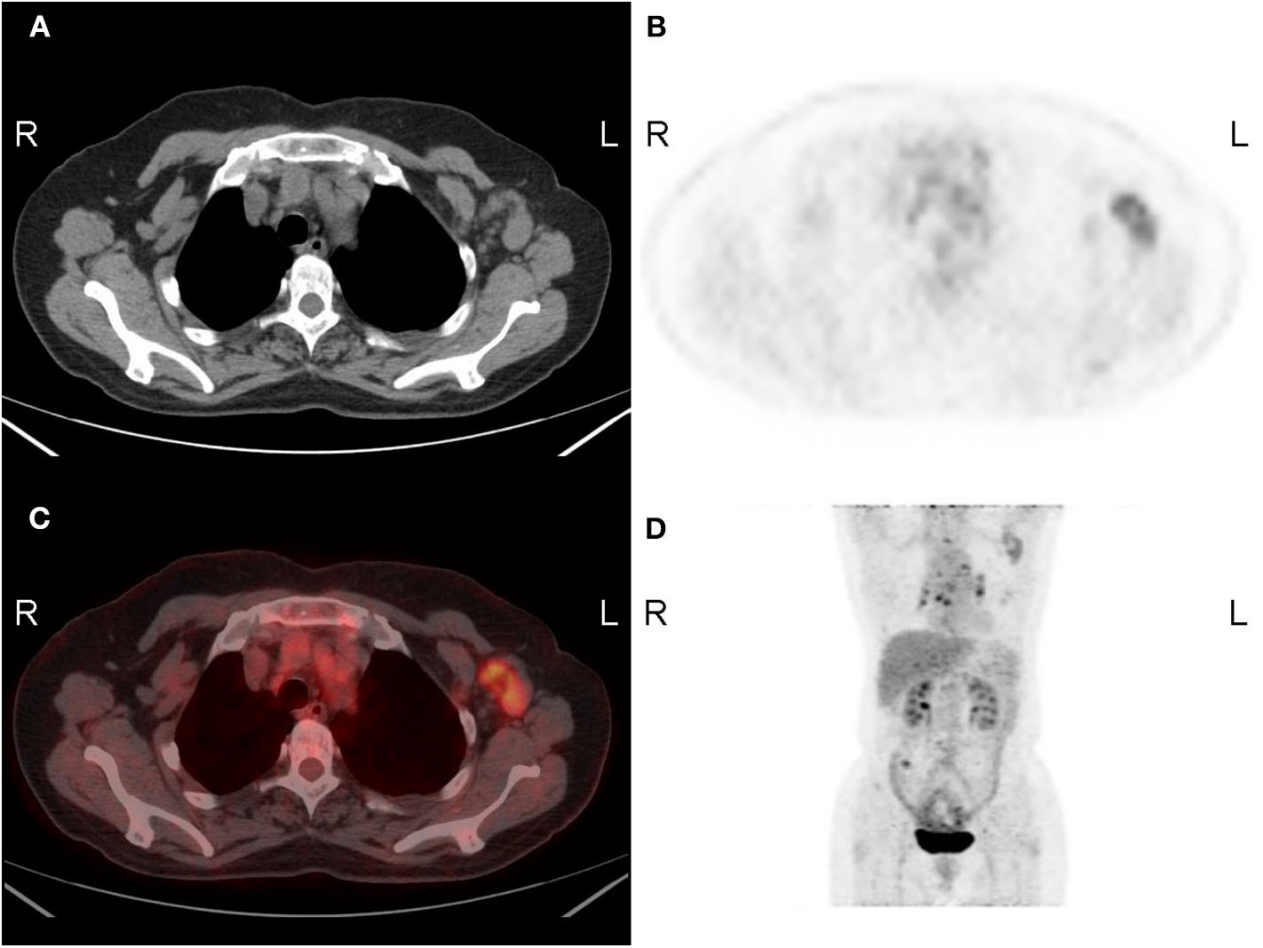
A 66-year-old woman with lymph-node metastasis of the left breast. Axial **(A)** CT, **(B)** FDG-PET, **(C)** fused image, and **(D)** MIP image show enlarged lymph nodes with intense uptake in the left axilla. The SUVmax was 3.4. R, right; L, left.

Although sentinel lymph node (SLN) mapping and biopsy are routinely applied to evaluate lymph-node metastases of breast cancer, imaging techniques are utilized as additional modalities in nodal assessment. Most studies have indicated that PET-based imaging is more sensitive in detecting axillary metastases than MRI (30). Figure 3 shows an example of the imaging lymph-node metastasis in a 66-year-old female patient with right breast cancer by PET/CT and MRI. Ergul et al. (45) reported that the sensitivity of PET/CT in detecting axillary metastasis was 67%, while that obtained from MRI was 47% (*N* = 15). The specificity of PET/CT for detecting axillary nodal involvement was also higher than that of MRI (89% by PET/CT vs. 78% by MRI, respectively). In a study of 128 patients with IDC, the c-statistic of predictive axillary lymph node (ALN) metastasis achieved a score of 0.791 when using a PET/CT-based model (46). It is worth noting that the CT portion of PET/CT appears to be critical for the sensitivity of axillary state detection. It has been reported that the sensitivity of PET alone for axillary metastases is 60%, while that of MRI alone is 93.3%, and the specificity for axillary metastases is 91% for both PET alone and MRI alone (47). In a study of 49 breast cancer patients (18 with axillary metastases), the sensitivity for axillary lymph-node status was 78% for PET/CT, 78% for PET/MR, and 67% for MRI, while PET/CT also demonstrate a slightly superior specificity of 94% for axillary metastases, compared with 90% from PET/MRI and 87% from MRI (34). In a feasibility study, van Nijnatten et al. (48) reported a dedicated axillary FDG PET/MRI that improved sensitivity to 100% (*N* = 12) in axillary nodal staging for clinically positive patients. Thus, compared with MRI, PET/CT and PET/MRI both show largely advanced accuracy in ALN detection.

Generally, ^18^F-FDG PET/CT is not recommended for axillary staging, especially in patients with small tumors, as PET/CT entails the delivery of a relatively high radiation dose (49). ^18^F-FDG-PET displays moderate sensitivity for axillary metastases that is insufficient for eliminating the necessity for sentinel lymph-node biopsy (50). The high positive predictive value of PET-based techniques allows surgeons to submit axillary node-positive patients directly to axillary lymph-node dissection (ALND) (51). In a study conducted in the Netherlands, 159 patients underwent combined FDG-PET/CT and MARI (marking axillary lymph nodes with radioactive iodine seeds) procedures for axillary staging. According to the results, most of the patients received direct locoregional radiotherapy without ALND. Subsequently, the authors reported an 82% reduction of ALND for node-positive breast cancer patients (52).

On the other hand, FDG-PET is helpful in identifying unsuspected lymph nodes in addition to ALNs that cannot be effectively assessed during SLN biopsy. Studies have consistently shown that ^18^F-FDG-PET is superior to CT in detecting internal mammary (IM) and mediastinal lymph-node metastases (53). A study of 58 breast cancer patients compared the performance of PET/MRI and MRI alone in the detection of axillary, internal mammary, and supraclavicular lymph nodes. For IM and supraclavicular lymph-node detection, the difference between PET/MRI and MRI was not statistically significant (31). Jochelson et al. (54) also found a similar detection rate of IM adenopathy by MRI and PET/CT (14/90 [16%] for MRI, 13/90 [14%] for PET/CT, *P* = 0.317). In general, PET/CT, MRI, and PET/MRI hitherto appear equivalent in the detection lymph-node metastases located in less common sites.

In brief, emerging studies have reported the fine detection power of PET-based techniques in lymph-node staging, but improvement and further studies are still necessary with regard to clinical utility. We deem that PET/MRI might become favored, especially in identifying unsuspected lymph nodes, due to its low radiation dose.

### Imaging Distant Metastases

Although PET-based imaging is not a standard procedure in staging early-stage breast cancer, it plays an important role in systemic staging. Groheux et al. (55) showed that PET/CT detected unsuspected metastases in up to 47.1% of patients with untreated stage III breast cancer. The upstaging rates reported for each stage were similar among subtypes of estrogen receptor [ER]+/HER2– and HER2+ breast cancer (56). The bone, liver, and lung are the most common sites of the distant metastasis of breast cancer (57). Generally, PET/MRI exhibits higher sensitivity than PET/CT in detecting metastatic lesions, especially in the liver and bones, but it is unable to detect lung metastasis (58–60). Moreover, PET/CT is thought to have low sensitivity for brain (61, 62) and liver (63) metastases.

Compared with whole-body CT and bone scintigraphy, ^18^F-FDG-PET can detect early bone marrow metastases before the signs of bone abnormalities appear on CT or bone scintigraphy (64). CT and bone scintigraphy cannot separate active or therapy-burnout lesions, so the prognosis from these techniques can be ambiguous. For example, bone metastases initially presenting as osteolytic lesions on CT could appear as osteosclerosis as a result of the repair process and become morphologically abnormal after successful chemotherapy or the introduction of bisphosphonates. On the other hand, increased ^18^F-FDG uptake indicates active bone metastasis regardless of the morphological appearance of the bone and can be accurately detected with PET-based imaging (65, 66). Furthermore, PET/CT can be helpful in predicting subsequent pathological fracture in metastatic breast cancer patients (67). Using F-FDG/F-NaF cocktail tracers, Roop et al. (68) reported significant advancement in bone metastasis detection by PET/CT. Despite the advantages of PET/CT in bone metastasis detection, PET/MRI demonstrated an improved performance (69). Catalano et al. (70) reported that PET/MRI could identify significantly more bone metastases than PET/CT in breast cancers (*N* = 25, *P* < 0.001).

In the liver, PET/MRI imaging depicted increased sensitivity compared with PET/CT (80–100% by PET/MRI vs. 70–75% by PET/CT, *P* < 0.001), which might be due to the application of DWI. Regarding pulmonary metastases, in contrast to bone and liver metastasis, PET/CT was reported to be slightly superior to PET/MRI in a study of 242 distant-metastatic lesions (59). Nevertheless, most of the lung lesions omitted by PET/MRI were small (<1 cm) or determined on follow-up, and thus, the clinical importance is unclear (71).

### Imaging Local Recurrence

PET-based imaging is of great use in the detection of tumor recurrence and unexpected remote metastasis in patients with local recurrence (72). In a study of 23 female patients with suspected breast cancer recurrence undergoing both PET/CT and whole-body MRI, PET/CT demonstrated higher specificity and sensitivity in the assessment of nodal and distant lesions than MR/DWI but lower sensitivity in the detection of local breast lesions (73). The overall sensitivity, specificity, and accuracy of PET/CT were 84.8, 86.3, and 85.4%, respectively, vs. 82.1, 78.0, and 80.5% for MRI (73). Additionally, PET/CT appeared to show an advantage over CT and PET alone in the diagnosis of breast cancer recurrence (74). Impressively, dual time-point PET/CT yielded an area under the ROC curve of 0.99 for recurrence detection (*n* = 100) (75). Imaging features of lymph nodes, rather than those of primary tumors, were shown to be even better predictors of recurrence (76). When combined with percutaneous FDG-avid target biopsies, PET/CT achieved a sensitivity of 99% for local recurrence surveillance (77). Compared with standard practice, PET/CT may offer improved diagnostic accuracy in breast cancer recurrence. It is also recommended to perform FDG-PET/CT at the same time as diagnostic CT in the prediction of breast cancer recurrence.

Whole-body PET/MRI is comparatively unfavored for detecting local recurrence. In a study of 94 primary breast cancer patients, all primary breast tumors were identified by prone breast/MRI, while whole-body PET-MRI missed primary breast cancers in 7/94 (7.4%) patients (78). Regarding small tumors, Kong et al. (27) demonstrated that only 4/10 (40%) breast cancer tumors <1 cm in length were seen on whole-body PET/MRI. Additionally, the contrast-enhanced MRI at 3T was demonstrated more sensitive in detecting small lesions (<10 mm) (79). Thus, PET/MRI is considerably sensitive to lesions in other parts of the body, and breast MRI or prone breast PET/MRI might be superior to supine whole-body PET/MRI in detecting local recurrences.

### Imaging the Therapeutic Response

Predicting the therapeutic response to chemotherapy in breast cancer is another important application of PET-based techniques. High-dimensional features in PET, CT, and MR images can be extracted with radiomics (80). Serial PET imaging after treatment is helpful for therapy response monitoring (81, 82). Changes in ^18^F-FDG uptake induced by different therapies might be valuable signs in the separation of responsive and non-responsive patients in an early phase (83). The therapeutic response is also deemed a powerful prognostic stratification indicator (for both disease-free survival and overall survival) in either locally advanced breast cancer or metastatic breast cancer (84, 85).

NAC has been proven to be substantially effective in primary tumor pre-surgery downstaging and is now increasingly utilized in stage II and III breast cancer (86). Abundant studies have confirmed the efficacy of PET/CT in the early assessment of the response to NAC. However, this method of assessment has not been introduced in routine clinical practice. In a study of 23 patients with locally advanced breast cancer, Kumar et al. (87) reviewed that the sensitivity, specificity, and accuracy of PET/CT in response detection were 93, 75, and 87%, respectively. Tian et al. (88) conducted a meta-analysis encompassing a total of 22 studies including 1,119 patients, and the pooled data showed that the sensitivity and specificity were 81.9 and 79.3%, respectively, for PET/CT in predicting the pathological response to NAC in breast cancer patients. In another meta-analysis of 19 studies involving 920 pathologically confirmed patients (89), the pooled sensitivity and specificity of PET in the prediction of the histopathological response in primary breast lesions were 84 and 66%, respectively. Additionally, in regional lymph nodes, the sensitivity of PET was 92% (89). Based on PET/CT parameters, the concept of complete metabolic response (CMR) has been introduced as a promising outcome predictor during the mid-course of NACs (90).

The treatment response in metastatic breast cancer could also be predicted by a quantitative assay based on changes in tracer uptake throughout the treatment. Groheux et al. reported that changes in ^18^F-FDG uptake (defined as ΔSUV_max_) are highly associated with the pathological complete response (pCR) after NAC (*P* = 0.0001) (91). Reported by two studies, a high SUV decrease after the first cycle of chemotherapy was able to predict the clinical response at the end of the treatment (92, 93). Couturier et al. (94) also reported that the changes in SUVmax after three cycles of chemotherapy could predict not only the clinical response but also the overall survival.

In a comparison of the performance of PET and MR imaging in predicting the pathological complete response (pCR), PET appears more sensitive, and MR imaging is more specific (88). Integrated PET/MRI metric data and clinical features can help improve accuracy. PET/MRI imaging characteristics can include changes in SUV_max_, total lesion glycolysis (TLG), signal enhancement ratio (SER), and peak enhancement ratio (PER) in pilot studies. Clinical data, namely, patient age, and breast cancer subtype, have also been taken into consideration (95–97). Additionally, MRI-derived anatomical data such as DWI and DCE sequences can also improve the specificity and negative predictive value of PET in response prediction (98).

The diagnostic performance and favorable factors of PET/CT and PET/MRI in breast cancer are compared in Table 1. For primary breast cancer, PET/MRI performed similarly to MRI but better than PET/CT. PET/CT and PET/MRI have higher sensitivity than MRI in the detection of axillary lymph-node metastases. However, in the detection of sentinel lymph nodes (SLNs), the sensitivity of the PET-based imaging methods was lower. PET-based imaging has shown its value in detecting unsuspected extra-axillary nodal metastases. For distant metastasis, PET/CT performs better in the detection of lung metastasis, while PET/MRI performs better in the liver and bone. Additionally, PET/CT is superior in terms of monitoring local recurrence. However, only a few cancer centers are equipped with a PET/MRI device, which severely limits the clinical application of the technique.

**Table 1:** Diagnostic performance of PET/CT and PET/MRI in breast cancer.

**Lesion**	**PET/CT**	**PET/MRI**
Primary breast cancer	Low	Moderate
Lymph node		
Axillary	High^*^	High^*^
Extra-axillary	High	High (favored)
Metastasis		
Liver	Moderate	High
Bone	High	High (favored)
Lung	High	Low
Local recurrence	High	Moderate

* *Lower than SLN biopsy*.

## Recent Advances in PET-Based Strategies

### Special Populations

Despite the medical concern for women who are at high risk for breast cancer, PET-based techniques have scarcely been used in cancer screening in this population due to the lack of solid evidence (99). Although genetic high-risk features can also be investigated using PET imaging as a result of the various changes in normal tissues (8), it is relatively difficult to assess the true utility of the technique. *BRCA1* mutation carriers have a higher risk of developing triple-negative breast cancer (100), which might appear with significantly increased ^18^F-FDG avidity (14). Therefore, compared to standard screening techniques, PET breast cancer screening might show improved sensitivity and a better chance of clinical utility in patients with *BRCA1* mutations, but further investigations are needed.

### Novel Radiotracers

Various new radiotracers are being developed and applied for the *in vivo* measurement of different aspects of breast cancer, such as proliferation, metastasis, hypoxia, receptor status, tumor antigen levels, and therapeutic response. Herein, we summarized some of the newly developed radiotracers (Table 2). As a cellular proliferation marker, ^18^F-fluorothymidine (^18^F-FLT) allows the *in vivo* investigation and quantification of tumor growth and metastases and is also utilized in identifying the treatment response (101, 102). ^18^F-Fluoromisonidazole (FMISO), which is a hypoxia biomarker, has also been used in breast cancer imaging since the degree of hypoxia in a tumor is associated with its biological features, such as aggressiveness and therapeutic resistance (6). Integrin αvβ3 overexpression appears in endothelial cells activated by tumor-induced angiogenesis and has been reported to be a marker of local advancement and metastasis of breast cancer (6, 125, 126). The ^18^F-galacto-arginine-glycine-aspartic acid tripeptide, which can bind to integrin αvβ3, has been used as a PET tracer to measure αvβ3 expression in both primary tumors and metastatic lesions (125). ^68^Ga-PSMA, which is a tumor-specific antigen that was first used in prostate cancer staging, has been repeatedly utilized and is rapidly becoming a complementary tracer for breast cancers with low FDG avidities. High PSMA uptake has been exhibited in invasive ductal carcinoma, triple-negative breast cancer, and rare pathological subtypes such as Signet-ring cell breast adenocarcinoma (110–112, 127). Like ^18^F-FDG, tracers based on cellular metabolism are continuously being studied. ^18^F-Fluciclovine, which is a leucine analog, has been shown to be strongly correlated with the tumor response after NAC therapy (106). ^18^F-Choline PET/CT has been reported to be efficacious in leptomeningeal metastasis detection (113).

**Table 2:** Novel radiotracers being studied in breast cancer.

**Tracer**	**Target mechanism**	**Applications**	**References**
18F-fluorothymidine	Thymidine analog	Tumor proliferation imaging	(101, 102)
18F-FMISO	Hypoxic cells	Tumor hypoxia imaging	(6)
68Ga-NOTA-RM26	Targeting GRPR	ER+ tumor detection of proliferation phase patient	(103)
68Ga-BBN-RGD	Targeting GRPR and integrin αvβ3	Primary tumor and metastases detection, especially ER+ tumor	(104)
68Ga-NOTA-RGD	Targeting integrin αvβ3	Angiogenesis imaging, recurrence prediction and prognosis prediction	(105)
18F-Fluciclovine	Leucine analog	Primary tumor and metastases detection, NAC response prediction	(106–109)
68Ga-PSMA	Targeting tumor-specific antigen	TNBC and ASRC detection	(110–112)
18F-Fluorocholine	Cell membrane component	Primary tumor and metastases detection	(113, 114)
68Ga-NO2AP-BP	Macrocyclic chelator	Skeletal metastases detection	(115)
89Zr-trastuzumab	Targeting HER2	HER2+ tumor detection	(116)
64Cu-DOTA-trastuzumab	Targeting HER2	HER2+ primary tumor and metastases detection	(117, 118)
89Zr-Pertuzumab	Targeting HER2	HER2+ primary tumor and metastases detection	(119)
68Ga-ABY-025	Targeting HER2	HER2+ tumor detection	(120)
18F-FES	Targeting ER	ER+ tumor, endocrine therapy monitoring and prognosis prediction	(121–123)
68Ga-DOTATATE	Targeting somatostatin receptor	Exclusion of fibroadenoma	(124)

*TNBC, triple-negative breast cancer; ASRC, signet ring cell breast adenocarcinoma; GRPR, gastrin-releasing peptide receptor; FES, 16α-17β-fluoroestradiol; FMISO, fluoromisonidazole; NO2AP-BP, triazacyclononane bisphosphonate*.

Radiotracers targeting hormone receptors and HER2 are also being investigated and may eventually help in the development of new drugs and in the evaluation of their efficacy (128–132). ^18^F-16α-Fluoroestradiol (FES) is a substrate of estrogen receptors. It has been used as a tracer for specific receptors in breast cancer and has been proven to be significantly associated with ER expression (133). Studies have indicated that FES avidity can be a pharmacodynamic biomarker for ER-directed therapy (121, 122). Another novel tracer, ^68^Ga-NOTA-RM26, was found to be correlated with both ER expression and menstrual status and to improve the sensitivity and specificity of breast cancer diagnosis to 100 and 90.9%, respectively, in proliferating phase patients (103). HER2 expression status plays an important role in prognosis and chemotherapy in breast cancer; trastuzumab and pertuzumab are two HER2 antagonists currently in use. In clinical practice, HER2 status is determined by immunohistochemical or fluorescence *in situ* hybridization testing of biopsy samples. Nevertheless, the test results might be affected by tumor heterogeneity and expression discordance between the primary tumor and metastasis sites (134). Therefore, new PET tracers such as ^89^Zr-trastuzumab and ^89^Zr-pertuzumab were developed for measuring the HER2 expression of the primary tumor and metastases simultaneously in a non-invasive manner and have shown promising results in clinical studies; nevertheless, false-positive foci still remain a challenge to their effective utility (116, 135, 136). In addition, ^68^Ga-labeled affibody molecules were shown to accurately measure HER2 expression in a phase I/II clinical trial of advanced breast cancers (137).

### PET Radiomics

Radiomics has the potential to uncover disease characteristics by extracting numerous parameters/features from tomographic images within a region of interest using mathematical algorithms (80). Non-invasive image-derived biomarkers can also be generated from PET radiomics according to the intensity of the pixels, their associated parameters, and their positions (138). Based on the clinical application of PET-based techniques, several studies have focused on the clinical and technical feasibility of applying PET radiomics to diagnosis (139, 140), staging (141–143), pathological characterization (17, 18), NAC response (18, 144–146), and outcome prediction (147, 148) in breast cancer. However, the difficulty in detecting the edges of breast lesions using CT limits the PET/CT in identifying the breast tumor and the alignment of the imaging modalities (141). As MRI has shown high sensitivity in breast cancer detection, combining PET- and MRI-derived radiomics features would further widen the spectrum of features and enable the building of more powerful predictive models (138, 142). However, most radiomics-based predictive models or classifiers have been found to be of insufficient quality because of the lack of complete information for model development (149), and they should be further validated with independent cohorts from external sources before application in clinical practice.

## Summary

To date, the development and clinical application of PET/CT and PET/MRI have shown their individual usefulness in breast cancer. This progress also presents opportunities to reclassify tumors by combining their fine anatomical features with metabolic characteristics and develop a beneficial pathway from bench to bedside to better understand the biological and pathophysiological bases of breast cancer. According to current clinical guidelines, PET-based imaging is not suitable for early-stage breast cancer in the absence of symptoms and is not indicated in the staging of clinical stage I or II or operable stage III breast cancer. However, PET/CT and PET/MRI add value in identifying regional nodal disease and distant metastases as an adjuvant to standard diagnostic imaging techniques. Nevertheless, further investigations are still necessary to optimize the detection power, efficiency, and predictability of PET/CT and PET/MRI. Although some studies have investigated the utility of PET-based strategies in high-risk women, current evidence does not support the use of PET as a screening technology. Moreover, novel radiotracers based on tumor biological behavior, hormone receptors, and HER2 status have emerged and will hopefully promote the precise detection of breast cancer. Recent advances in PET radiomics could further widen the spectrum of features, especially when combined with MRI-derived radiomics. Similar to other radiomics-based predictive models, independent validation of PET-based radiomics models in multiple centers are still needed before application in clinical practice. We conclude that comprehensive and convergent applications of PET approaches will become a future trend in the clinical management of breast cancer. With the expansion of tumor information provided by advanced examination techniques, the treatment of breast cancer can further develop via greater individualization and precision.

## Author Contributions

All authors listed have made a substantial, direct and intellectual contribution to the work, and approved it for publication.

## Conflict of Interest

The authors declare that the research was conducted in the absence of any commercial or financial relationships that could be construed as a potential conflict of interest.
